# Rectenna System Development Using Harmonic Balance and S-Parameters for an RF Energy Harvester

**DOI:** 10.3390/s24092843

**Published:** 2024-04-29

**Authors:** Muhamad Nurarif Bin Md Jamil, Madiah Omar, Rosdiazli Ibrahim, Kishore Bingi, Mochammad Faqih

**Affiliations:** 1 Department of Chemical Engineering, Universiti Teknologi PETRONAS, Seri Iskandar 32610, Malaysia; nurarif.jamil@utp.edu.my (M.N.B.M.J.); madiah.omar@utp.edu.my (M.O.); mochammad_22000035@utp.edu.my (M.F.); 2 Department of Electrical and Electronics Engineering, Universiti Teknologi PETRONAS, Seri Iskandar 32610, Malaysia; rosdiazli@utp.edu.my

**Keywords:** RF energy harvesting, rectenna, Wi-Fi, low RF power

## Abstract

With the escalating demand for Radio Frequency Identification (RFID) technology and the Internet of Things (IoT), there is a growing need for sustainable and autonomous power solutions to energize low-powered devices. Consequently, there is a critical imperative to mitigate dependency on batteries during passive operation. This paper proposes the conceptual framework of rectenna architecture-based radio frequency energy harvesters’ performance, specifically optimized for low-power device applications. The proposed prototype utilizes the surroundings’ Wi-Fi signals within the 2.4 GHz frequency band. The design integrates a seven-stage Cockroft-Walton rectifier featuring a Schottky diode HSMS286C and MA4E2054B1-1146T, a low-pass filter, and a fractal antenna. Preliminary simulations conducted using Advanced Design System (ADS) reveal that a voltage of 3.53 V can be harvested by employing a 1.57 mm thickness Rogers 5880 printed circuit board (PCB) substrate with an MA4E2054B1-1146T rectifier prototype, given a minimum power input of −10 dBm (0.1 mW). Integrating the fabricated rectifier and fractal antenna successfully yields a 1.5 V DC output from Wi-Fi signals, demonstrable by illuminating a red LED. These findings underscore the viability of deploying a fractal antenna-based radio frequency (RF) harvester for empowering small electronic devices.

## 1. Introduction

The advent of the Internet of Things (IoT) has profoundly transformed our interaction with the contemporary world, giving rise to a myriad of battery-dependent electronic devices. The utilization of batteries, however, introduces issues related to degradation, wherein the internal chemical compositions undergo continuous reduction and oxidation processes during multiple charging cycles and elevated temperature operations, leading to a decline in capacity over time and usage [1]. The constant replacement of batteries in small devices becomes a cumbersome task, mainly when these devices are situated in locations that are distant from human interaction or remote areas. Consequently, there is a discernible trend in adopting alternative power solutions to energize low-powered devices. One such application is Radio Frequency Identification (RFID), which interfaces with electromagnetic fields to identify and locate tags affixed to objects such as cars, parcels, and sensors. Projections indicate a substantial growth trajectory for RFID technology in the global market until 2033 [2].

In an initial case study, Selim and Wu [3] engineered an optimized voltage multiplier comprising two matched single-stage multipliers. Their findings indicated that the rectifier achieved a harvesting capacity of 3.51 V and 3.51 mA at an applied power of +7 dBm, coupled with an applied load of 1 kOhm and a maximum efficiency of 66%. The study also demonstrated the ability to harvest 12.34 mW of power at the maximum power input (Pin) of +20 dBm, operating at a frequency of 2.45 GHz. A subsequent investigation by Mathur, Agarwal, et al. [4] resulted in developing a seven-stage rectifier with a coplanar monopole antenna. This configuration yielded an output of 289 mV with an applied power of −26.2 dBm. The harvested antenna power was measured at 5 µW, and the antenna exhibited resonance within the frequency range of 900 MHz to 9.9 GHz, with a band rejection from 3.1–5.6 GHz. However, the prototype’s efficiency was reported at 25%. A third study conducted by Sangaran, Ramasamy, and Din [5] focused on the design of a prototype for low-power sensors and mobile-charging applications. Their 8-stage Villard RF energy harvesting system, equipped with a custom-built antenna and power management circuit, demonstrated the capability to harvest a DC output voltage of 0.35 V within the Wi-Fi band at 2.4 GHz and 185 cm from the source. Integration with a BQ25570 power management system resulted in a regulated output of 3 V.

Adam, Yasin, et al. [6] developed a 6-stage Dickson topology rectifier, operating within the frequency band of 2.4 GHz. Utilizing a 1.6 mm thickness FR-4 substrate with a permittivity of 4.7, the prototype generated 3.4 V with 0 dBm input power. Notably, the harvester illuminated a green LED at 5 dBm input power. However, the prototype exhibited limitations, failing to harvest above 1.5 V at power inputs below −10 dBm. In a study made by Ipar, Lambor, and Joshi [7], a patch antenna and RF-harvesting module were developed. The prototype demonstrated harvesting capabilities of 35 mV indoors and 31 mV outdoors, both under a signal strength of −10 dBm after a 10-min charge. A supercapacitor integrated into the harvester module allowed the harvested energy to power devices such as a calculator, LED, or a 1.2 V battery.

The collective data showcasing the potential of RF harvesters in powering low-energy devices provides an optimistic impetus for further prototype development. Thus, the contributions of this manuscript are as follows:

- Optimization of cascading 7-stage Cockroft–Walton rectifier design with the implementation of an MA4E2054B1-1146T Schottky diode through harmonic balance simulation and prototype experimentation, forecasting an efficiency increase and, therefore, an improvement in the capability of harvesting DC output at lower power input at 2.4 GHz frequency with 1.5 V benchmark;
- Simulation of S-parameters, along with realization and performance test of fractal antenna design as a viable component in electromagnetic radiation in the form of microwaves into electrical energy conversion;
- Contribution in quantitative analysis of the potential and performance of the energy-harvester prototype with a rectifier and fractal antenna combination in capturing radiation signals from a Wi-Fi source in a real environment.

Section 2 of this paper presents the rectenna architecture, while Section 3 encompasses the design and simulation of the rectifier and fractal antenna. Section 4 details the simulation results, data interpolation, and ensuing discussion, and Section 5 consolidates the outcomes of this work, outlining future directions.

## 2. Rectenna Architecture

**Figure 1:**
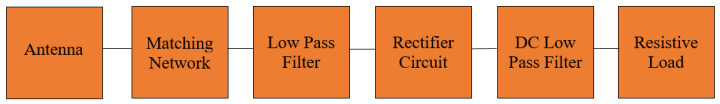
Common rectenna architecture.

A rectenna combines a receiving antenna fusion and a rectifying circuit to transform microwave energy into direct current (DC) power [8]. Illustrated in Figure 1, a prevalent rectenna configuration comprises an antenna, a matching circuit, a low-pass filter (LPF), a rectifying circuit, a post-rectification low-pass filter for the DC path, and a resistive load.

In a standard rectenna architecture, the placement of a low-pass filter directly after the matching network is a strategic design choice aimed at optimizing the energy-harvesting process. The matching network serves a critical function by ensuring that the impedance of the antenna is matched to that of the rectifier circuit, maximizing the power transfer and minimizing reflection losses. Following the matching network, the low-pass filter plays a pivotal role by suppressing any higher-order harmonics or noise that could be generated by the antenna or the matching process.

After the low-pass filter, the signal is conveyed to the rectifier circuit. The rectifier’s primary function is to convert the filtered RF signal, which is alternating current (AC), into a direct-current (DC) signal. This is typically achieved through the use of diodes or transistors that only allow current to flow in one direction, effectively converting the AC waveform into a pulsating DC waveform.

Following rectification, the output still contains residual AC components. To smooth these fluctuations and obtain a more stable DC output, the signal is passed through a DC low-pass filter. This filter is specifically designed to remove the ripple by only allowing frequencies below a certain cutoff point. The result is a smoother DC signal.

The filtered DC signal is then delivered to the resistive load. In practical applications, the resistive load represents the end-user device or storage mechanism for the harvested energy. The quality of the DC power delivered at this stage is crucial, as it directly affects the performance and efficiency of the end-user application. The more stable and smooth the DC output, the more effective the power utilization, ensuring the rectenna’s efficiency and the reliability of the power supply to the load.

### 2.1. Rectifier Topology

**Figure 2:**
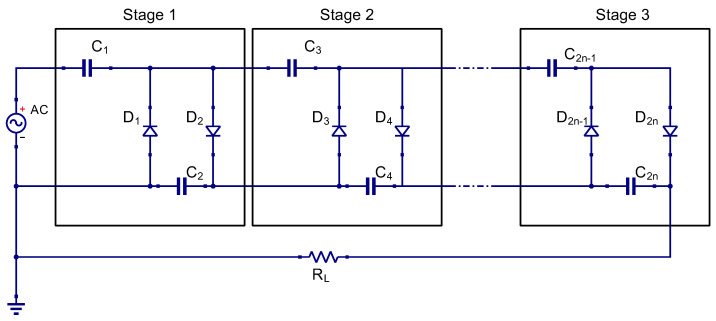
Circuit diagram of Cockroft–Walton topology design.

The investigation for a suitable candidate capable of stable operational conditions involves the assessment of Cockroft–Walton, Dickson, and Greinacher rectifier circuit topologies [9]. Each stage within these topologies comprises a parallel configuration of two capacitors and two diodes, as depicted in Figure 2 [10]. According to Adam, Yasin, et al., the cascading of multiple stages of Schottky diodes can result in a high DC voltage output, provided the diodes exhibit a low forward voltage threshold. However, implementing an excessive number of stages is limited due to the potential reduction in impedance caused by accumulating parasitic capacitances at high frequencies, leading to a consequent decrease in harvested output voltage [11].

In the context of low-input RF power operation, the optimum selection of diode parameters is paramount for the efficient functioning of the voltage multiplier. Consequently, the diode is chosen based on minimizing junction capacitance and series resistance, especially during low-input RF power operation [12]. Several models are considered to identify the Schottky diode with the highest conversion efficiency, with the results summarized in Table 1. Notably, the HSMS 2860 diode demonstrates superior conversion efficiency [13]. However, its availability is limited to the single-configuration small-outline transistor (SOT-23). This study chooses the HSMS286C diode, commonly employed in a series pair configuration (SOT-323), for design efficiency. Despite its discontinuation, the MA4E2054B1-1146T Schottky diode is introduced as a viable replacement, offering a high series resistance of 11 Ω and a lower junction capacitance of 0.13 pF compared to the HSMS286C.

The selection of a seven-stage Cockroft–Walton half-wave voltage rectifier circuit is grounded in its simplicity and uncomplicated design. This topology affords greater flexibility in enhancing the number of stages. Additionally, the Greinacher Topology incorporates twice the number of diodes as the Cockroft–Walton, potentially leading to heightened parasitic losses and a consequent reduction in DC output voltage [13].

The study in Table 2 shows the energy harvested with two substrate materials of different RF input power and configurations. Substrate RO5880 with 1.57 mm thickness shows a promising performance as the ratio conversion of power input to DC output is higher than in other studies.

The operational frequency for the envisioned RF harvester is situated within the 2.40 GHz band. Therefore, the selection of capacitance is influenced by the high-frequency operation, where the preference lies in employing fast-switching capacitors to attain maximal output voltage. At each stage of the rectifier, a 1uF capacitor is employed to uphold low impedance from the RF source to the load, as documented in [17]. This capacitance was selected to yield a nearly constant DC output voltage, guided by the ripple voltage formula presented in Equation (1):

(1) $$V_{r}=\frac{V_{M}}{2fRC}$$

In the above equation, $V_{r}$ represents the ripple voltage, $V_{M}$ is the peak voltage, *f* is the frequency of operation, *R* denotes the resistive load, and *C* signifies the capacitance value [18]. The attainment of a small ripple voltage is contingent upon the RC time constant being substantially greater than the frequency range in the gigahertz (GHz) domain. Additionally, a singular 1nF capacitor is positioned at the output capacitance or close to the load, enhancing transient response, as outlined in [19].

### 2.2. Harmonic Rejection Filters

The rectification process exhibits pronounced nonlinearity, primarily attributable to the presence of diodes. Consequently, effective mitigation of high-frequency harmonics engendered by the rectifiers necessitates filtration. By attenuating higher-order harmonics beyond the fundamental frequency, the extraction of output power is facilitated [20]. Concurrently, with the augmentation of output power, a corresponding elevation in output voltage is achieved. Equation (2) delineates the correlation between power (P) and root mean square (RMS) voltage (VRMS) within the context of a standard 50-Ohm radio-frequency system, using a reference wattage of 1 mW [21]:

(2) $$P\left(dBm\right)=20\log\left(\frac{V_{RMS}}{0.224}\right)$$

This study applies a 2.40 GHz band pass filter (BPF) for pre-rectification. At the same time, post-rectification employs a combination of passive components, such as an inductor and capacitor integrated into a microstrip configuration. This specific implementation suppresses higher harmonics, thereby augmenting the additional output DC voltage, a methodology validated by Kim [22]. Consequently, Kim’s approach is incorporated into the present work. Notably, these filters can be realized using surface-mount devices (SMDs), offering the advantage of significantly reducing filter dimensions on the printed circuit board (PCB) while adhering to specified operating frequencies [23].

### 2.3. Transmission Line Modeling

The microstrip transmission line serves as a conduit for power transfer, accommodating both DC and AC signal propagation. In RF design applications, a standard trace width of 12 mils or 0.3048 mm is commonly employed [24]. The design of microstrip line structures adheres to the criterion that the ratio of trace width *w* to dielectric thickness *h* falls within the specified range of 0.1 < w/h < 3.0. Consideration is given to the impact of discontinuities in the design process [25]. For the transmission line configuration made in ADS, the models utilized include Microstrip Line (MLIN), Libra Microstrip T-Junction (MTEE-ADS), and Optimally Chamfered Bend (MSOBND), which are assigned for modeling horizontal traces, vertical traces, junctions between tracers, and 90-degree bends, respectively. This selection aids in mitigating impedance mismatches between traces [26].

### 2.4. Substrate Laminate

Two material substrates, namely, the widely adopted FR4 and Rogers5880, are subjects of investigation concerning their impact on rectifier and antenna performances. These substrates’ dielectric constants and tangent losses are recognized as influential factors. A comprehensive performance comparison, as outlined in Table 2, reveals that the RO5880 substrate exhibits superior voltage-harvesting capabilities compared to FR4. Furthermore, the substrate choice of RO5880 features a low loss-tangent material characteristic over FR4. A lower loss tangent results in more transmitted signals. Moreover, the harmonic balance simulation on the rectifier design showed that the substrate RO5880 yielded better overall DC output when compared with the FR4 substrate. Consequently, the RO5880 substrate emerges as the preferred choice for rectifier design. As for the antenna substrate, both FR4 and RO substrate are considered and simulated with HFSS software. S-Parameters preliminary simulation showed that the antenna with FR4 substrate shows the best −10 dB bandwidth response at frequencies near to 2.4 GHz compared to RO5880, hence making it the choice of material for antenna fabrication.

### 2.5. Matching Network

An issue that arises in rectifier design is that the input power from RF sources is not directly proportional to the rectified output voltage. At low power input, the rectifier system is unable to proportionately rectify the voltage. However, after considerable increase in input power, the system can rectify transiently. The non-linearity is due primarily to the forward voltage threshold from the Schottky diode. For the used modeling of the diodes, the forward voltage from the specification sheet has values between 250 mV and 350 mV. This results in the Schottky diode being unable to rectify the DC voltage output of the cascading rectifier system. Moreover, the parasitic behavior of the diodes and other electronic components reduces the efficiency of the rectifier. To optimize the non-linearity of the output, a matching circuit can be implemented in the design. A matching circuit, an integral facet of antenna and rectifier systems, is critical in facilitating efficient power transfer between the transmission line. The primary objective of the matching circuit lies in matching the impedance characteristics of the antenna and rectifier with those of the transmission line, conventionally expressed as 50 Ω in the context of most RF (radio frequency) systems. The importance of impedance matching cannot be overstated, as it constitutes a pivotal factor in optimizing power-transfer efficiency while concurrently minimizing signal reflections. Instances where the impedance of the antenna diverges from that of the transmission line can yield deleterious effects such as signal loss, diminished operational efficiency, and a Voltage Standing-Wave Ratio (VSWR). A high VSWR indicates inefficient power radiation by the antenna, accentuating the importance of precise impedance matching [27]. Matching circuits, encompassing components such as inductors, capacitors, and transformers, necessitate a tailored design contingent upon the inherent impedance characteristics of the antenna, the rectifier, and the transmission line. The overarching goal is the precise adjustment of impedance to align with the transmission line’s characteristic impedance. This alignment serves as the linchpin for efficient power transfer and the amelioration of signal reflections. The matching circuit emerges as an indispensable constituent within the systems, which is pivotal in optimizing impedance alignment between the system and the transmission line. This optimization, crucial for ensuring the efficacy of radio-frequency signal transmission and reception into the rectifier, underscores the significance of meticulous attention to impedance matching in rectenna system design.

### 2.6. Fractal Antenna

Antennas exhibit diverse shapes and sizes, so achieving optimal performance at specific frequencies often requires larger dimensions. To integrate with the small form factor of rectifier dimension, a microstrip-fed hybrid fractal antenna, designed to cater to WLAN applications, is presented, aiming to reconcile the challenge of achieving efficient operation within constrained size requirements [28]. In his investigations, Jamil introduced an antenna configuration of meander and Koch elements, ingeniously combined to form an elongated curve, effectively augmenting the electrical length. This unique approach achieves a dual objective of reducing both the operational frequency and the overall dimensions of the antenna. Green’s functions and the segmentation method are employed to meticulously calculate its input impedance for a thorough analysis of the hybrid fractal antenna. The intricate geometry of the combined antenna is systematically dissected into simpler regular segments, facilitating a comprehensive analysis. The impedance parameters of these segmented shapes are computed using corresponding Green’s functions. The resultant impedance matrix equations, encompassing all segments, are then consolidated into a singular matrix. The solution to these equations intricately determines the input impedance for the entire antenna geometry. In simulations and measurements conducted by Jamil, the antenna exhibited gains of 2 dBi and 1.9 dBi at the frequency of 2.4 GHz.

## 3. Design and Fabrication

### 3.1. Schottky Diode Parasitic Model

**Figure 3:**
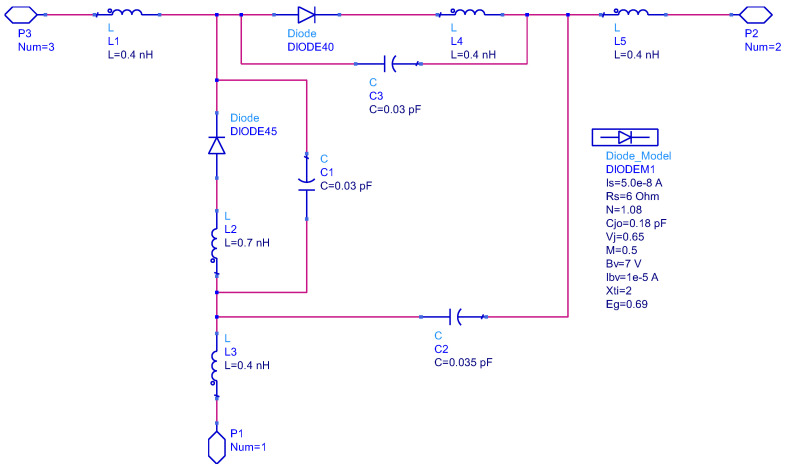
Parasitic model of HSMS286C Schottky diode.

**Figure 4:**
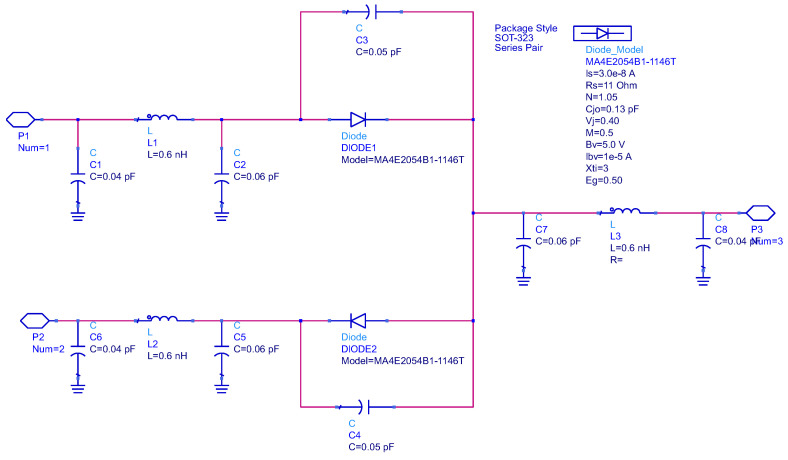
Parasitic model of MA4E2054B1-1146T.

The Schottky diode is systematically characterized through adherence to the manufacturer’s specification datasheets and through incorporating a Simulation Program with Integrated Circuit Emphasis (SPICE) parameters. Specifically, a series pair configuration of the HSMS286C diode and MA4E2054B1-1146T diode is implemented within the SOT-323 package. The corresponding models are visually represented in Figure 3 and Figure 4.

### 3.2. Seven-Stage Rectifier and Bessel Low-Pass Filter Design

**Figure 5:**
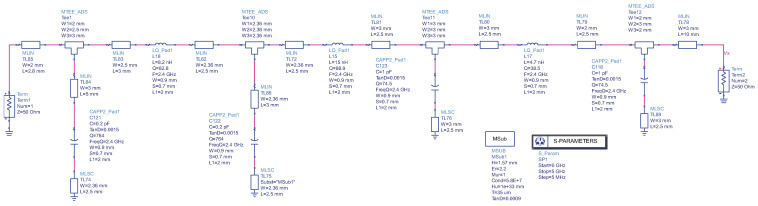
Bessel low-pass circuit diagram.

The decision to adopt the Bessel low-pass filter (LPF) design is grounded in Kikkert’s model, illustrated in Figure 5 [29]. The cut-off frequency is tailored to yield a −3 dB attenuation at 2.45 GHz, employing a 7th-order Bessel LPF. Subsequently, a thorough S-Parameter simulation is executed to analyze and document the response characteristics of the filter.

**Figure 6:**
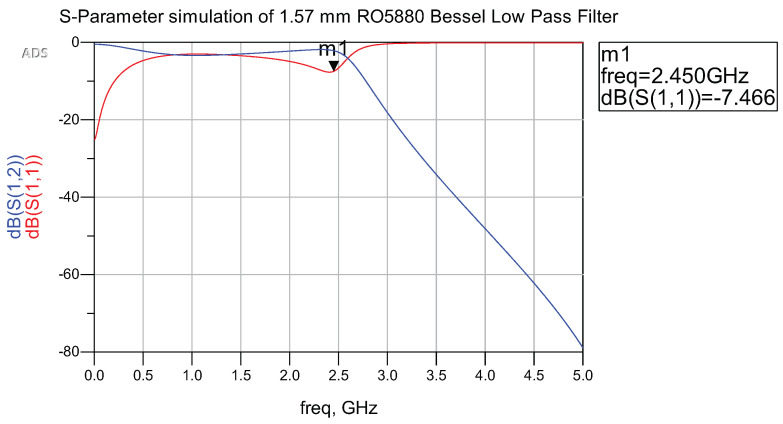
Bessel low-pass filter performances.

Simulated performance of S-Parameter is presented in Figure 6. The outcomes reveal successful attenuation of frequencies beyond 2.45 GHz, affirming the intended performance of the Bessel low-pass filter. The reflection coefficient performance is the most effective at 2.45 GHz, with a magnitude of −7.466 dB, which denotes effective rejection of higher frequencies. Consequently, integrating the low-pass filter circuit with the rectifier for rejecting unintended high frequencies has been achieved.

The design of the seven-stage rectifier involves the cascading of seven one-stage rectifiers, each comprising an embedded Schottky diode, two capacitors, and interconnected transmission lines. The Schottky diode is modeled as a sub-circuit and is positioned within the primary circuit. Subsequent fine-tuning of passive components and transmission lines is conducted within the rectifier and low-pass filter to optimize for maximum output and to achieve high efficiency.

**Figure 7:**
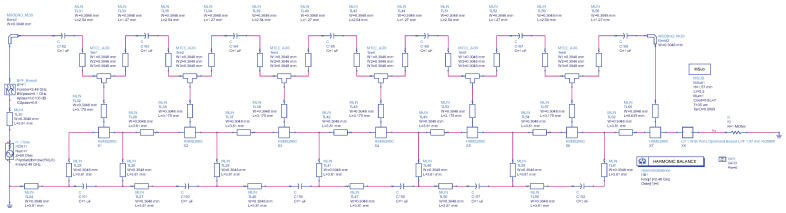
Optimized 7-stage rectifier with BPF and LPF.

To ascertain the steady-state solution of the nonlinear circuit and output voltage, the harmonic balance simulation method is employed to assess the performances of the diodes [30]. The input and output are configured with a 50 Ω impedance, and the input frequency is set at 2.4 GHz to emulate power reception from an antenna. The substrate utilized is RO5880 from Rogers, serving as the PCB board. The layouts are designed and simulated using the Advanced Design System (ADS), as shown in Figure 7.

**Figure 8:**
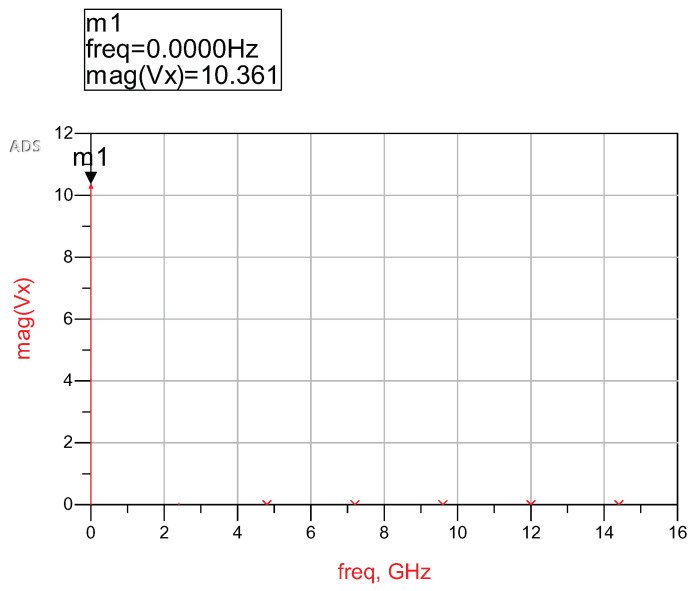
Magnitude of spectrum from harmonic balance simulation of 7-stage rectifier with BPF and LPF.

The depiction in Figure 8 presents the magnitude spectrum derived from the harmonic balance simulation of the 7-stage rectifier, incorporating BPF and LPF, at an input power level of 0 dBm. A noteworthy observation is that, at the frequency point of 0 GHz, where no oscillating frequency is evident in the DC output, this results in a magnitude of 10.361 V. The subsequent simulation involves a systematic sweep of input power, ranging from −20 dBm to 0 dBm, to examine the pattern in the DC output.

Table 3 presents the simulated voltage output derived from harmonic balance simulations conducted with diverse configurations. Improved rectifiers are simulated with 1.57 mm, whereas standard rectifiers are simulated with 0.787 mm RO5880 from the identical RO5880 substrate. The escalation in rectifier stages substantiates a concurrent increase in output voltage. Notably, the simulation outcomes ascertain that an eight-stage rectifier exhibits superior performance compared to its seven-stage counterpart, aligning with the observations reported by Hong et al. Implementation of a seventh-order LPF manifests an augmentation in the output DC voltage. Furthermore, augmenting the dielectric thickness from 0.787 mm to 1.57 mm yields a discernible improvement in the output DC voltage. Evaluating the impact of various Schottky diodes in a one-stage rectifier reveals marginal advancements in output DC voltage. However, a comprehensive analysis involving a seven-stage rectifier with LPF and distinct Schottky diodes underscores the superior performance of MA4E2054B1-1146T Schottky diodes over HSMS 286C. The culmination of optimized components and a judicious filter layout for the seven-stage rectifier, coupled with the implementation of MA4E2054B1-1146T Schottky diodes, establishes an optimal configuration that yields the highest achievable output voltage.

**Figure 9:**
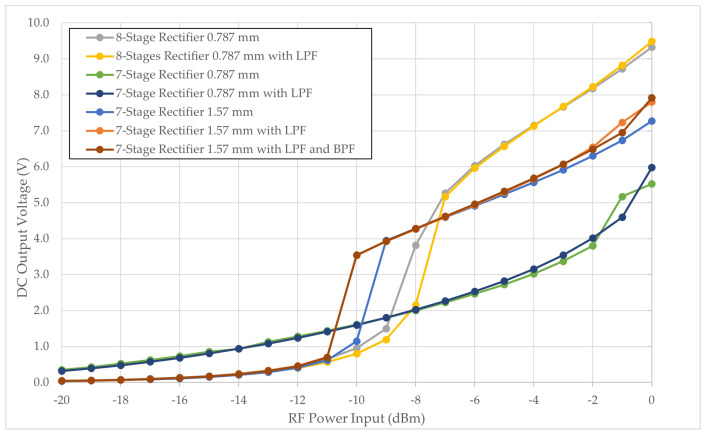
Simulated DC output voltage from HSMS286C Schottky diode configuration.

The simulation implemented designs employing the HSMS286C diode, incorporating a power sweep input ranging from 0 to −20 dBm. The outcomes of this simulation are illustrated in Figure 9. The 7-stage rectifier with a 0.787 mm dielectric thickness demonstrates a consistent increase in output as the power input ascends, with a notable distinction observed at power inputs of −11 dBm and beyond. In contrast, the 8-stage rectifier design with a 0.787 mm thickness manifests the highest harvested output at elevated RF power inputs, while the 7-stage rectifier with 1.57 mm design exhibits the capacity to extract DC voltage at lower RF inputs, albeit not reaching the levels observed in the 8-stage design. The transient surge in DC voltage occurs at −11 dBm for 1.57 mm rectifiers, −10 dBm for 7-stage 1.57 mm, and −9 dBm for 8-stage rectifiers, suggesting the necessity of a higher RF power input to overcome the parasitic capacitance threshold. The term parasitic capacitance threshold refers to the inherent limitation of the rectifier design, where the parasitic behavior of Schottky diodes, capacitors, and inductors affect the overall performance. Overcoming the parasitic capacitance threshold implies that the rectifier design needs to be optimized to minimize the effects of these parasitic elements. The similar behavior of parasitic capacitance effect discovered in previous related work [31], where such surges were noted at power input levels of −15 dBm and below. This indicates a discernible shift in circuit performance at this juncture. Our current observations echo these findings, albeit with the transient phenomena occurring at different input levels, suggesting a similarity in underlying electrical mechanisms. The correlation underscores a consistent pattern, reinforcing the significance of the parasitic capacitance threshold in dictating the performance boundaries of rectifier circuits in RFID applications.

The 7-stage rectifier with a 1.57 mm substrate thickness is the optimum condition. Introducing an LPF and BPF into the circuit reduces the minimum RF power input required for the rectifier. However, as the RF power input increases, there is minimal discernible power output difference among all three configurations. Compared to the rectifier with LPF and standalone rectifier configurations, the rectifier with BPF and LPF yields a slightly higher output.

**Figure 10:**
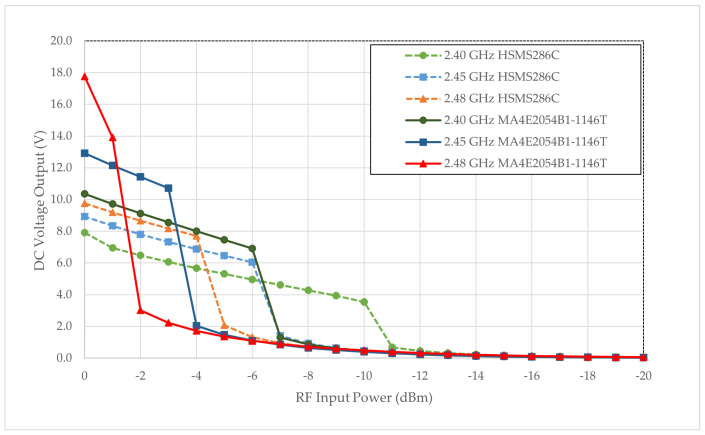
Simulated DC output for 7-stage rectifier with different Schottky diodes on 3 frequency bands.

Figure 10 delineates the performance evaluation of the 7-stage rectifier, featuring a BPF and LPF, employing an HSMS286C Schottky diode and MA4E2054B1-1146T across three discrete frequencies of 2.40 GHz, 2.45 GHz, and 2.48 GHz. The substrate maintains a constant thickness of 1.57 mm throughout the analysis. While all configurations manifest an escalating trend in harvested DC voltage with increasing RF input power, the rectifier employing the MA4E2054B1-1146T model exhibits suboptimal performance at lower input power levels. Still, it showcases improvement at higher RF input power than its HSMS286C counterpart.

Under the frequency of 2.40 GHz, the HSMS286C diode achieves a DC voltage of 1.5 V as early as −10 dBm, while the MA4E2054B1-1146T model yields 1.3 V at −7 dBm. At elevated RF input power of 0 dBm, the MA4E2054B1-1146T rectifier attains a maximum output of 17.76 V. Nevertheless, a minimum output of 0.30 V is achievable when the RF power input is approximately −11 dBm for all frequency bands and configurations. Based on the simulation results, the rectifier prototype utilizing the MA4E2054B1-1146T model is selected for subsequent fabrication.

**Figure 11:**
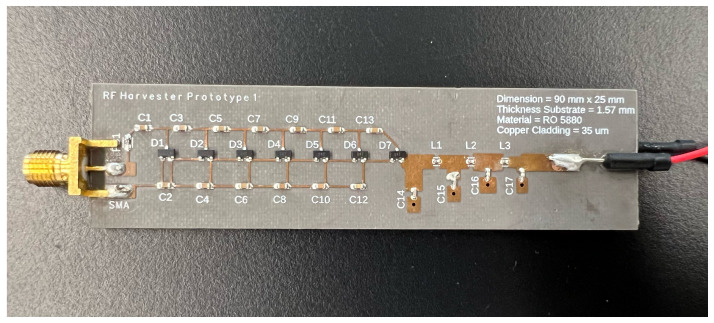
Fabricated 7-stage rectifier with BPF and LPF.

Initially formulated in ADS software, the circuit design is translated to Fusion360 software for enhanced fabrication efficiency. The upper surface of the rectifier encompasses the intricacies of the circuit design, while the bottom layer features a copper layer, serving the essential function of a ground plane, as depicted in Figure 11. Integration of the DC output wire and an SMA female connector jack occurs in subsequent stages post-fabrication. The first and second prototype rectifiers are fabricated with HSMS286C and MA4E2054B1-1146T, respectively.

### 3.3. Fractal Antenna

The fractal antenna design draws inspiration from the research conducted by Jamil et al. The principal objective of the proposed antenna geometry is to elongate the convoluted curve, thereby reducing the resonance frequency. This goal is realized by amalgamating the meander line and Koch curve elements in an iterative approach to shape the antenna.

The construction of the hybrid meander-Koch curve fractal antenna commences with a linear strip divided into five equal segments. Subsequently, a rectangular element is introduced at the center, giving rise to a meander element that occupies 3/5 of the total strip length. This meander structure encompasses two vertical lines and a horizontal line. Over this, the Koch curve element is superimposed, achieved by dividing the horizontal line into three segments and replacing the middle part with two lines inclined at angles of 45° and −45°, respectively.

In the subsequent phase of the geometric transformation, the identical two-level procedures are systematically applied to each linear segment until the ultimate geometry of the meander-Koch curve antenna is achieved. This iterative process involves transforming an initiator (represented by a straight line) using a generator created by the combination of meander and Koch curve geometries. The fractal transformations are defined by the subsequent equation [32]:

(3) $$W\left[\begin{array}{c} x\\ y \end{array}\right]=\left[\begin{array}{cc} \frac{1}{r}cos\theta & -\frac{1}{s}sin\theta \\ \frac{1}{r}sin\theta & \frac{1}{s}cos\theta \end{array}\right]\left[\begin{array}{c} x\\ y \end{array}\right]+\left[\begin{array}{c} e\\ f \end{array}\right]$$

where the parameters *r* and *s* scale the segments in the *x*- and *y*-directions, respectively. θ represents the rotation angle, and the column vector (e,f) performs translations along the *x*- and *y*-directions, respectively.

**Figure 12:**
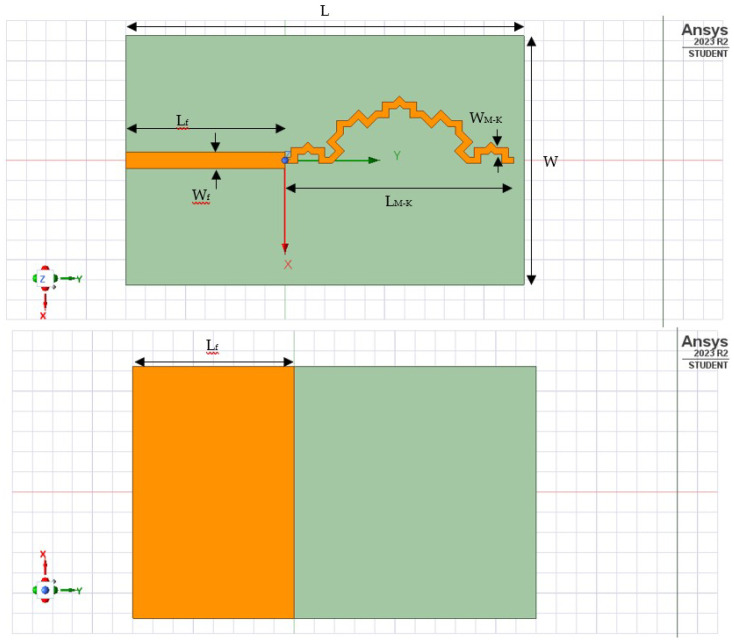
Top and bottom layer of fractal antenna design.

The antenna design process is executed using Ansys Student Desktop HFSS to simulate return loss (S_11_) measurements and the Voltage Standing-Wave Ratio (VSWR). Following the simulation phase, the design is physically fabricated on an FR4 substrate with specific material properties, where the dielectric constant $\epsilon_{r}$ = 4.3 and the board thickness h = 1.6 mm. The precise dimensions of the fractal antenna are outlined in the accompanying Table 4, and the layout design is shown in Figure 12.

### 3.4. Return Loss S_11_ (dB) and Voltage Standing-Wave Ratio

In the assessment of rectenna system performance, both return loss and the Voltage Standing-Wave Ratio (VSWR) serve as critical indicators of impedance-matching effectiveness. The parameter S_11_ holds significance as a return loss or reflection coefficient metric. S_11_ quantifies the proportion of RF energy reflected due to impedance mismatches within a transmission line or network. Mathematically, S_11_ is expressed as the ratio of impedance of reflected b_reflected_ to incident of power waves a_incident_ when a signal is transmitted into a device or network [33,34]:

(4) $$S_{11}=\frac{b_{\mathrm{reflected}}}{a_{\mathrm{incident}}}$$

The incident and reflected power waves *a*_incident_ and *b*_reflected_ are defined in Equations (5) and (6):

(5) $$a_{\mathrm{incident}}=\frac{V_{1}+Z_{1}\times I_{1}}{2\sqrt{|ReZ_{1}|}}$$

(6) $$b_{\mathrm{reflected}}=\frac{V_{1}-Z_{1}\times I_{1}}{2\sqrt{|ReZ_{1}|}}$$

*V*_1_ and *I*_1_ are the voltage and the current flowing into the one-port junction of the rectifier system and *Z*_1_ is the impedance exiting from the one-port with real positive impedance at the square root of the denominator.

(7) $$S_{11\mathrm{dB}}=20\log\left|S_{11}\right|$$

Often presented in decibels (dB), S_11_ can be articulated as shown in Equation (7). A low S_11_ value or a high negative dB value suggests minimal reflection and an effective impedance match, fostering efficient power transfer in RF systems. Since S_11_ is a complex number, any deviation from zero—whether it presents as complex, real and positive, or real and negative—indicates the occurrence of reflections. It signals a potential impedance mismatch at any state, including when port resonance is established.

**Figure 13:**
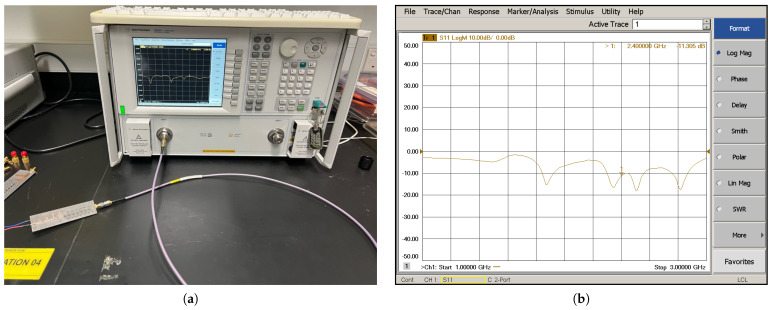
Measurement of return loss performance of rectifier. (**a**) Second rectifier prototype experiment setup with PNA E8363C Network Analyzer. (**b**) S_11_ response of second rectifier prototype.

**Figure 14:**
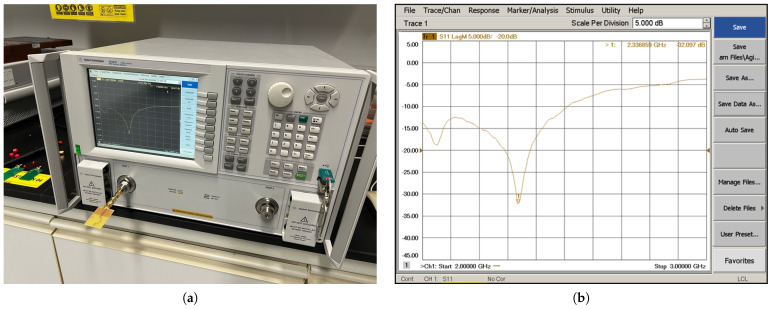
Measurement of return loss performance of the fractal antenna. (**a**) Setup for return loss measurement of fractal antenna prototype. (**b**) S_11_ response of fractal antenna prototype.

The measurement procedure was conducted utilizing a PNA E8363C Network Analyzer. The test probes to evaluate return loss comprised prototypes of the two rectifiers and a fractal antenna. The prototypes were linked to the analyzer through 50 Ω cables, while a direct connection of the antennas to the RF input of the analyzer was established to preempt any potential sensitivity alterations. The frequency band was systematically swept from 2 GHz to 3 GHz. The obtained measurements were meticulously recorded and organized in tabular form. The experimental arrangements are visually shown in Figure 13a and Figure 14a, whereas the S_11_ responses are depicted in Figure 13b and Figure 14b.

Similarly, VSWR is a crucial metric in radio-frequency (RF) engineering, employed to assess power-transfer efficiency between a transmission line and its associated load. This parameter serves as an indicator of the impedance match between the transmission line and the connected load. Mathematically, VSWR is defined as the ratio of the maximum voltage $V_{\max}$ to the minimum voltage $V_{\min}$ along the transmission line:

(8) $$\mathrm{VSWR}=\frac{V_{\max}}{V_{\min}}$$

where $V_{\max}$ represents the maximum voltage amplitude and $V_{\min}$ denotes the minimum voltage amplitude. The values of VSWR provide valuable insights into the degree of impedance matching. A VSWR of 1 signifies a perfect match, indicating minimal reflection.

In RF system optimization, pursuing lower VSWR values minimizes signal reflections and enhances overall power-transfer efficiency. The reflection coefficient is derived from S_11dB_ simulated and measured rectifiers and fractal antenna prototypes. As a result, VSWR can be determined.

### 3.5. Signal Strength of Fractal Antenna

**Figure 15:**
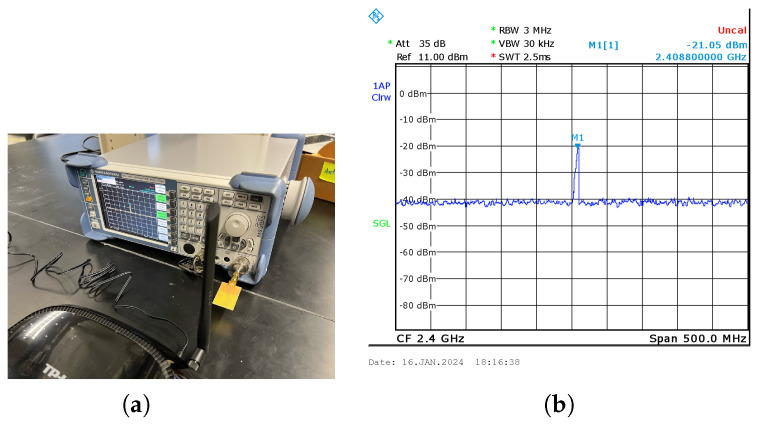
Measurement of signal strength of fractal antenna capturing energy from Wi-Fi power source. (**a**) Experimental setup of fractal antenna with Rohde and Schwarz FSL Spectrum Analyzer and Wi-Fi router. (**b**) Signal strength of fractal antenna at 15 cm distance from Wi-Fi router.

A spectrum analyzer is extensively utilized in the realms of electronics and telecommunications for the pivotal role of analysis of signal-frequency content. This instrument proves indispensable for precisely measuring and visualizing the frequency domain characteristics inherent in diverse electrical signals. In the experiment setup, the Rohde and Schwarz FSL Spectrum Analyzer is deployed to quantitatively assess the magnitude of an input signal emanating from the fractal antenna, as shown in Figure 15a. This assessment spans the entirety of the frequency spectrum encompassed by the instrument, offering valuable insights into the potential harvesting power associated with the antenna’s signal reception capabilities. The transmitting element in this scenario is a TP-Link Wi-Fi router model TD-VG3631, and the receiving antenna is directly interfaced with the RF input of the spectrum analyzer. These antennas are deliberately positioned in perpendicular alignment. Operating at a frequency of 2.4 GHz, the Wi-Fi router transmits a signal that the receiving antenna captures, with the signal strength displayed on the analyzer interface as shown in Figure 15b. As the distance between the Wi-Fi router and the receiving antenna increases, a consequential decrease in signal strength is observed. The experiment rigorously measures the strength of the antenna at systematic 5 cm intervals, contributing to a comprehensive understanding of signal behavior under varying spatial conditions.

#### 3.5.1. Direct Input with RF Generator DC Output Measurement

**Figure 16:**
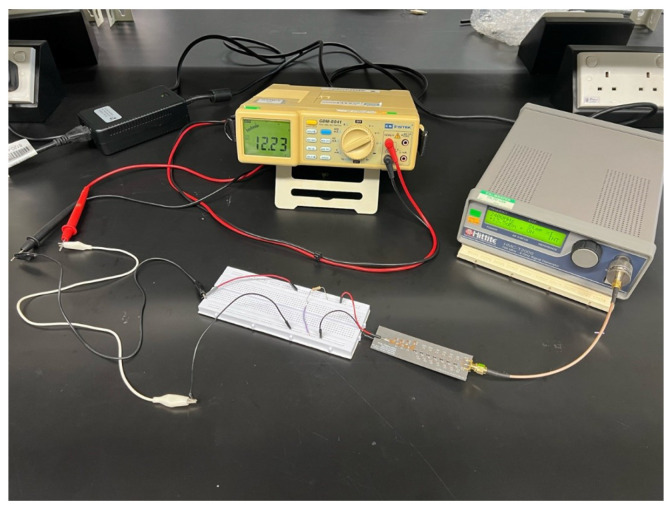
Setup measurement for controlled RF power input for resistor and LED load.

This section investigates the rectifier’s performance under varying RF input power conditions. A Hittite radio-frequency generator delivers a continuous RF power signal, allowing for adjustable frequency and power input settings, as shown in Figure 16. The prototype is connected directly to the RF generator using a 50 Ω cable and an adapter. A 1 MΩ resistor is incorporated into the DC output circuit. Voltage measurements are performed using a multimeter, which is connected across the resistor in the DC output circuit to accurately capture the voltage output. The frequency is set at 2.4 GHz, commencing with an initial input power of −20 dBm. Subsequent voltage readings are recorded at 1 dBm increments, with the measurement process extending to 20 dBm. Observations indicate that the multimeter readings fluctuate consistently when the voltage reading ascends from 0.2 V to 0.4 V. Consequently, a stabilization period of 30 s is implemented following each adjustment of the power input to ensure a stable and reliable reading.

#### 3.5.2. Red LED Indicator Feasibility Test

**Figure 17:**
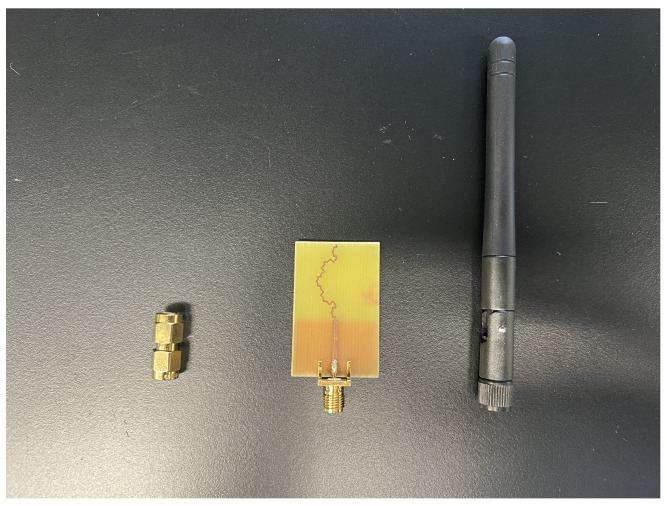
A male-to-male SMA connector jack, fractal antenna, and 2.5 dBi dipole antenna used for testing.

**Figure 18:**
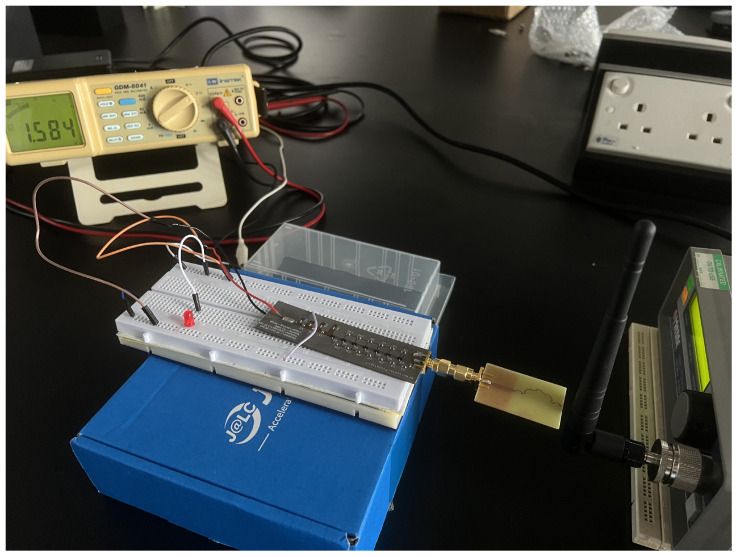
Rectenna setup testing under controlled RF power input.

A typical red LED would require 1.7 V to light up compared to other LED colours, which requires higher voltage and is, hence, suitable for this test [35]. Modifications are implemented in the experimental setup to assess the possibility of illuminating a red LED. Instead of directly connecting to the frequency generator, the rectifier is now linked to a fractal antenna with an adapter from Figure 17. The setup is illustrated in Figure 18. A readily available 2.5 dBi dipole antenna is attached to the frequency generator. The frequency is fixed at 2.4 GHz, and the power input is set to a maximum of 21 dBm (125.89 mW). The rectenna system gradually moves closer to the transmitting antenna, and the precise distance at which the red LED illuminates is recorded.

#### 3.5.3. DC Voltage Measurement of Rectenna System with Wi-Fi Router as RF Source

**Figure 19:**
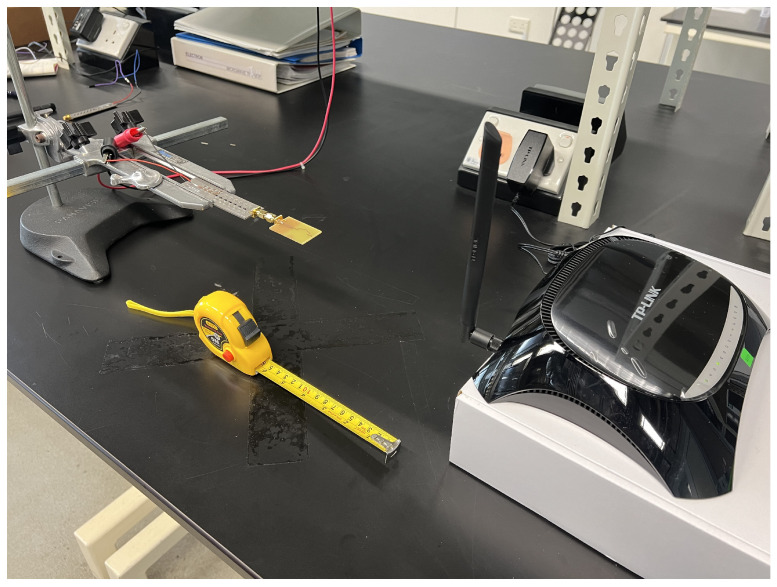
DC output voltage measurement of rectenna system with Wi-Fi router as RF source.

Further experimentation is undertaken, employing a Wi-Fi router as the RF power source instead of the conventional frequency generator. This approach aims to replicate real-world environmental conditions and assess the performance of the rectenna system accordingly. In this experimental configuration, a TP-Link TD-VG3631 model is designated as the power source, as depicted in Figure 19. The router is equipped with a single high-gain 5 dBi antenna, which is utilized for this setup. A GDM 8041 multimeter is intricately connected to the output of the rectenna system. Both antennas are precisely aligned at the same height and positioned in proximity. The Wi-Fi router is initiated at a fixed frequency of 2.4 GHz, and the voltage reading is initially recorded at a proximity of 0 cm. The distance between the router and rectenna is incrementally increased at 1 cm intervals, with careful recording of the multimeter readings. This procedure is systematically conducted until the distance between the rectenna and the Wi-Fi router reaches 30 cm.

## 4. Results and Discussion

This section examines the simulated and conducted experiments of return loss, VSWR, and harvested DC output of the prototypes. The quantitative values of the signal strength of the fractal antenna, voltage measurement from the rectifier, and rectenna system are briefly discussed.

### 4.1. Return Loss Results of the Two Rectifier Prototypes

**Figure 20:**
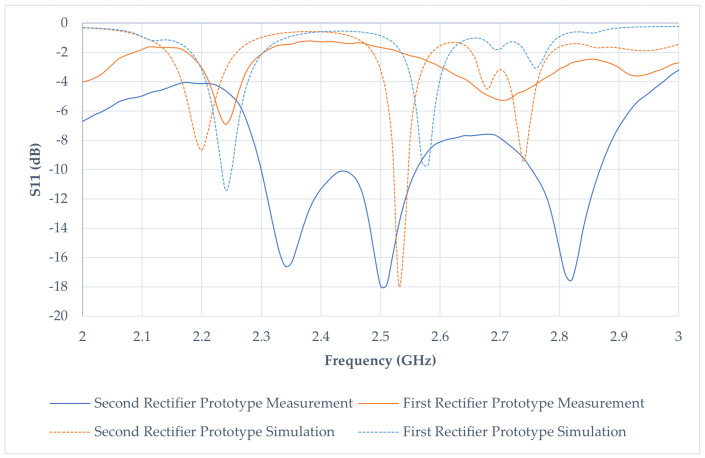
Reflection coefficient S_11_ simulation and measurement response from two rectifiers.

Figure 20 depicts the response characteristics of the initial and subsequent rectifier prototypes. The simulated S11 parameters indicate suboptimal performance for both prototypes. Specifically, the simulated S_11dB_ for the prototype, employing HSMS286C at 2.4 GHz, is recorded at −0.58 dB and is nearly congruent with the measured result at −1.28 dB. In contrast, the simulated result of the second prototype S_11dB_ at 2.4 GHz is −0.595 dB, displaying a considerable deviation from the measured value of −11.31 dB.

In a comparative assessment, the S_11dB_ of the second prototype surpasses that of the prototype, owing to its diminished return loss at 2.4 GHz. As mentioned earlier, a reduced return loss signifies a decreased reflection of incoming power to the rectifier, resulting in higher power transmission. This signifies a noteworthy advancement in the redesigned architecture relative to the initial prototype. Moreover, the second prototype exhibits commendable performance within the frequency spectrum of 2.30 GHz to 2.55 GHz, maintaining a return loss below −10 dB. This underscores the efficacy of the second rectifier across frequencies beyond 2.4 GHz, thereby indicating its potential suitability for deployment in alternative frequency bands.

Numerous parameters contribute to the irregularities observed in the S_11dB_ responses. Despite maintaining identical PCB boards and copper traces for both prototypes, discrepancies may arise in the capacitors, Schottky diodes, and inductors due to potential inaccuracies in their specifications resulting from fabrication errors. Significantly, Prototype 1 undergoes in-house fabrication, while Prototype 2 is outsourced to a vendor with state-of-the-art machinery and equipment. Consequently, the fabrication tolerance in the latter is considerably narrower, leading to minimal errors and optimal performance with minimal loss. This distinction underscores the critical role of fabrication processes and equipment precision in achieving enhanced performance and reduced variability in microwave device responses.

### 4.2. Standing-Wave Ratio of Rectifier Prototypes

**Figure 21:**
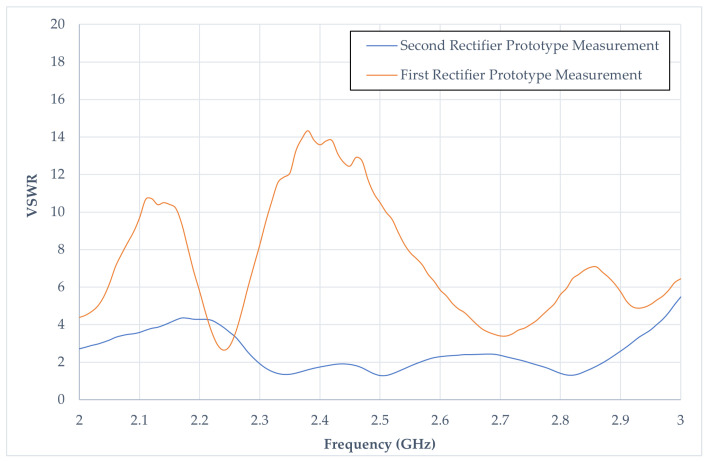
Voltage Standing-Wave Ratio response in two rectifiers.

Figure 21 illustrates the measured VSWR from the first and second prototypes. The VSWR of the first prototype exhibits a notably high ratio, displaying fluctuations across the frequency band ranging from 2.0 GHz to 3.0 GHz when compared to the second rectifier prototype. At the frequency of 2.4 GHz, the VSWR value for the first prototype stands at 13.59, while the second prototype records a significantly lower value of 1.57. The observed correlation between the VSWR values and the S_11dB_ results is noteworthy. A low VSWR ratio in prototype 1 signifies efficient transmission of power. In contrast, a high VSWR ratio indicates an elevated transmitted power reflection, resulting in a diminished harvest of DC output. As detailed in Table 5, the forward power in prototype 2 exhibits considerable improvement. In summary, the second rectifier prototype substantially enhances the first prototype, as evidenced by improved VSWR performance and superior forward-power characteristics.

### 4.3. S_11_-Parameters of Fractal Antenna Result

**Figure 22:**
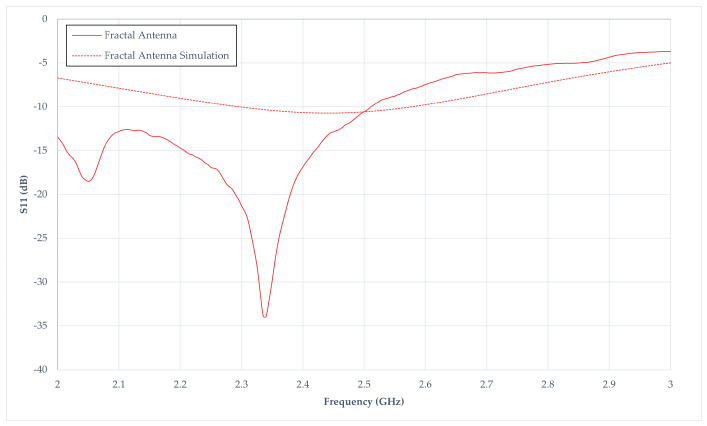
S_11_ results from fractal antenna simulation and measurement.

Figure 22 depicted the fractal antenna’s simulated and measured S_11_ performance. The simulated performance results in a marked deficiency at −10 dB. Specifically, at 2.4 GHz, the S_11dB_ records −10.66 dB, accompanied by a corresponding −10 dB bandwidth that spans 0.28 GHz (from 2.295 GHz to 2.575 GHz). In contrast, the response in the fabricated fractal antenna demonstrates favourable characteristics. The center frequency is situated at 2.34 GHz instead of 2.4 GHz. However, at 2.4 GHz, the S_11_ measures −16.87 dB, falling below the −10 dB range. The measured −10 dB bandwidth is extensive, ranging from 2 GHz to 2.51 GHz. Consequently, the fractal antenna exhibits suitability for broadband applications in energy harvesting.

Considerable disparities between simulated and measured results arise, potentially attributed to insufficient information in simulating the antenna. The intricate and time-intensive nature of the simulation process may contribute to this deviation. This discrepancy may be ascribed to imprecise measurements and calculations of the angle of the fractal antenna or impedances during simulation, impacting the precision of the simulated output. In contrast, the antenna fabrication is readily executable, aided by information from the works of Jamil et al., rendering it understandable and implementable with design software. Additionally, the discrepancies likely stem from conducting experiments in a real-world environment rather than an anechoic chamber, where environmental factors such as multipath reflections and interference can impact outcomes. Additionally, another factor such as the modification of wave-port connection and radiation boundaries should be investigated. The large discrepancies happened below the frequency of 2.4 GHz, but, beyond a frequency of 2.5 GHz, the experimental results begin to follow the simulation trend more closely. These large discrepancies between simulated and measured results suggested that further study is required in fractal antenna design for the future extension of this work.

### 4.4. Voltage Standing-Wave Ratio Measurement in Fractal Antenna

**Figure 23:**
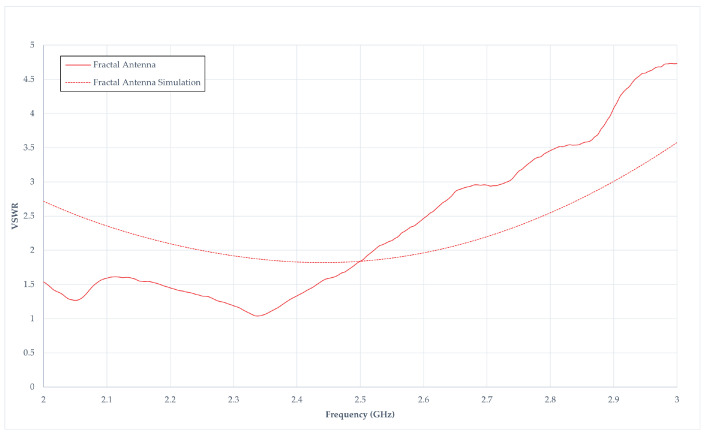
Voltage Standing-Wave Ratio responses in a fractal antenna.

Figure 23 illustrates the VSWR in the fractal antenna, showcasing both simulated and measured results. The simulated VSWR exhibits a distinctive curve, displaying a decrement from 2 GHz to 2.4 GHz, followed by an increase. Conversely, the measured VSWR reflects a decline from 2 GHz to 2.34 GHz, succeeded by a steady ascent with increasing frequency. At 2.4 GHz, the measured antenna’s VSWR is positioned at 1.33, while the simulated counterpart registers at 1.83. Both simulated and measured results manifest commendable VSWR ratios, indicative of efficient power transmission. The ideal scenario is achieved as the VSWR approaches 1, signifying 100% power transmission. However, despite a favourable VSWR ratio, Figure 23 reveals that the simulated S_11dB_ value suggests poor antenna effectiveness. Table 6 presents a comprehensive performance summary.

### 4.5. Result of Signal Strength of Antennas from Wi-Fi Router Power-Source Measurement

Table 7 provides an overview of the signal strength exhibited by the fractal antenna in capturing RF signals from a Wi-Fi router. The signal strength diminishes as the fractal antenna distance increases. Notably, the measured values between 5 cm and 30 cm yield an average of −21 dBm, indicating commendable performance exclusively within short ranges. According to the harmonic balance simulation for rectifiers, the anticipated theoretical voltage that rectifier prototypes 1 and 2 can sustain at very low power levels, ranging from −10 dBm to −20 dBm, is approximately 0.3 V on average. However, challenges may arise beyond −20 dBm power input as the rectification process becomes intricate due to the Schottky diode’s voltage threshold being inadequately designed for rectifying ultra-low AC voltage. This presents a hurdle for the rectifier in generating a substantial DC output. Further discussion on addressing this challenge will be incorporated in the subsequent improvement of the fractal antenna design.

### 4.6. DC Output Measurement of Rectifier with Direct Input Power from RF Generator

Table 8 elucidates the numerical results of the harvested DC voltage measured from rectifier prototypes 1 and 2, derived from a direct RF generator operating at a 2.4 GHz input frequency. Both prototypes exhibit a consistent and discernible linear decline pattern in measured output as the power input diminishes. The simulated rectifier is designed with high accuracies with pre-defined material characteristics, whereas measured results are subjected to tolerances of electronic components and transmission lines. Individual tuning of electronic components to its exact Q factor or TanD is challenging from manufacturer standpoint. Therefore, a compromise is made and, therefore, a strong deviation from the results.

**Figure 24:**
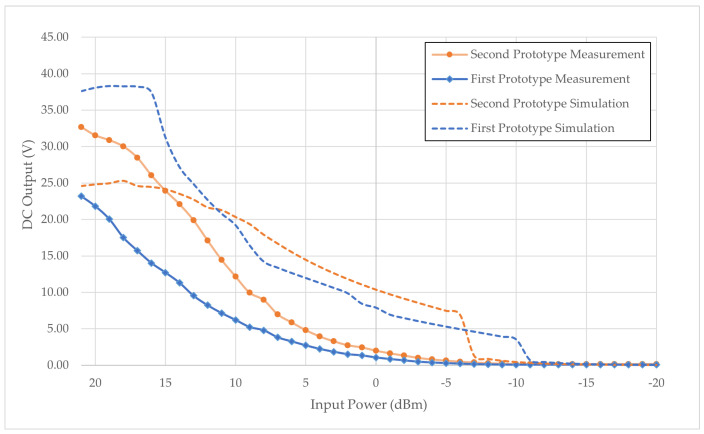
DC voltage output of rectifier prototype 1 and 2 with direct input power from an RF generator.

Upon scrutinizing the performance of prototype 1, the threshold of 1.5 V is achieved at a 2 dBm input with a 1 MΩ load, while, at 0 dBm input, this threshold is attained under a free load condition. In the case of prototype 2, the 1.5 V threshold is achieved at a −1 dBm input with a 1 MΩ load and −3 dBm input when unloaded. The results collectively indicate that both rectifiers necessitate an elevated power input to manifest the specified DC output of 1.5 V. Further comparative analyses between the measured rectifiers and their simulated counterparts are depicted in Figure 24.

The initial prototype simulation successfully yielded a voltage of 1.5 V at −10 dBm, while the second prototype simulation demonstrated an achievement of 1.5 V above −7 dBm. Both simulations exhibited a transient surge at the threshold, indicative of overcoming the low voltage threshold of Schottky diodes. Notably, the rectifying patterns observed in the simulations mirrored those obtained through measurements.

A discernible distinction emerged in the absence of a transient response and a lower output voltage in the measured results compared to the simulations. Furthermore, it was observed that the power input required for prototypes 1 and 2 to attain 1.5 V in the measured scenario exceeded that of the simulated counterparts. The harmonic balance simulation could not account for the parasitic behavior of the Schottky diodes within the seve-stage rectifier system, contributing to a substantial disparity between the simulated and measured DC outputs. The explanation of the deviation stems from initial parameters setup from the simulation and actual rectifier. The parasitic behavior input from the simulation is not identical to the actual values of the Schottky diodes, capacitors, and inductors.

An intriguing finding surfaced as the simulations indicated that, at an input power of 15 dBm, both prototypes reached their maximum rectifying capabilities and could not rectify further. However, an in-depth exploration of low-power inputs must be thoroughly investigated in the context of low-power applications, especially in environments with weak RF Wi-Fi signals. As the work progresses, optimizing the rectifier design and seeking enhanced performance from Schottky diodes are critical focuses for future developmental efforts.

### 4.7. Rectenna System Response with RF Generator

Table 9 presents the minimal distance necessary for the conjuncture of a rectifier and a fractal antenna to illuminate an LED with a power input of 21 dBm from an RF generator. For prototype 1, the distance covered is currently 11 mm, coinciding with the point when the red LED exhibits a faint glow, a notably brief span. In contrast, prototype 2 extends this distance by a mere 15 mm. Nevertheless, the accomplishment of illuminating a red LED at the 1.7–1.8V threshold is realized with the manufactured prototype. Both rectifiers are equipped with identical fractal antennas and connectors, affirming that, under the same RF to AC conditions, prototype 2 can harvest more energy.

### 4.8. Rectenna System Performance from Wi-Fi Power Source

The results of DC output measurement of the rectenna system with Wi-Fi are presented in Table 10. Notably, the 1.5 V threshold is attained within the 0 to 2 cm range. Specifically, 1 cm and 2 cm distances are essential for reaching the specified 1.5 V threshold. Within this range, the radiation strength of the Wi-Fi router is sufficiently robust for the fractal antenna to absorb the RF signals effectively.

Both prototype 1 and prototype 2 exhibit a negative exponential pattern, illustrating a decline in power input with increasing distance. Prototype 2 consistently maintains an average output of 0.3 V from 0 cm to 27 cm, while prototype 1 sustains an average output of 0.15 V until a distance of 10 cm is reached. This observation implies that while the 1.5 V threshold remains unattainable for both rectifiers at longer distances, they can maintain a particular voltage level over shorter distances. This finding aligns with the established correlation between the respective rectifiers’ VSWR and S_11_ values.

**Figure 25:**
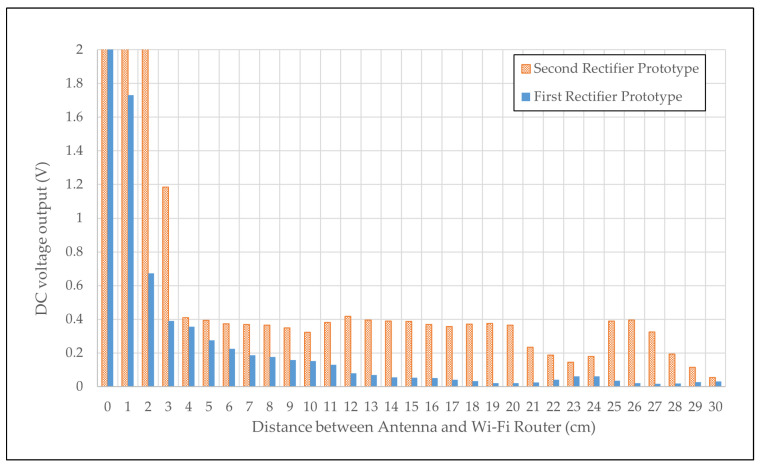
DC output measurement of rectenna system below 2 V threshold.

The illustration in Figure 25 delineates the harvested DC voltage from rectenna prototypes 1 and 2, specifically focusing on values below the 2 V threshold. This graphical representation unveils improvements in rectifier design when coupled with antennas. The observed trend in the measured DC output voltage for prototype 1 follows an exponential decline, stabilizing at an average output of 0.03 V within the distance range of 15 cm to 30 cm. In contrast, prototype 2 exhibits a sustained average output of 0.38 V up to a distance of 26 cm. Notably, the first rectenna can maintain a 1.5 V output at a mere 1 cm from the Wi-Fi router. In contrast, the second rectenna prototype achieves this at a slightly greater distance of 2 cm.

It is imperative to acknowledge that the continuous maintenance of 1.5 V, as projected by simulations for both rectifiers with a fractal antenna, still needs to be realized. This discrepancy between the measured and simulated results can be attributed to unforeseen limitations within simulation parameters, including the parasitic behavior of electronic components, which should have been considered during the simulation process. Tolerances within the fabricated electronic components introduce potential challenges, particularly in a cascaded rectifier design, where parasitic behavior or defects may accumulate, subsequently impacting the desired output.

### 4.9. Ambient Voltage Reading

Table 11 shows the ambient DC voltage reading from a multimeter when the rectenna protoype is not in proximity from a Wi-Fi router. Rectenna prototype 2 has an improvement, although marginal, compared to prototype 1. Although RF power at ambient environment can be harvested, the amount is too small to power a high-power IoT device instantaneously. The harvested energy can, however, be useful if the system is paired with an energy storage device such as a small battery, which can be charged over a long period of time. But, as for the scope of work in this paper, the conducted research is mainly on the passive condition of rectifier operation.

## 5. Benchmarking

This section compares the current work with previous works, as tabulated in Table 12. In the current work, the authors undertake a comprehensive evaluation of integrating fractal antenna technology with RF energy-harvesting systems, leveraging both simulation and experimental approaches. This stands in contrast to reference [36], which focuses on investigating rectenna architecture specifically for RFID applications through qualitative assessment, and reference [13], which examines rectenna architecture but places emphasis on various multiplier topologies to enhance the system for low-power applications.

The ultimate aim of the current work is to evaluate an integrated system comprising a fractal antenna and RF energy harvester through rigorous simulation and experimentation. The work achieves a measured output of 3.53 V at −10 dBm input, which is a considerable improvement in terms of efficiency over the outputs obtained in previous studies. For instance, reference [36] reports a 2 V output at a higher input power of −9 dBm using a similar frequency. The current work also outperforms reference [13], which achieves 2 V at −4 dBm input, and reference [9], which presents a 2 V output at −9 dBm.

In terms of rectifier configuration, the present study utilizes a seve-stage rectifier on an RO5880 substrate and integrates MA4E2054B1-1146T Schottky diodes, contrasting with reference [36]’s seven-stage rectifier with RO5880 substrate. Reference [13] employs multi-stage rectifiers designed for low power, while reference [37] and reference [9], respectively, focus on an efficiency-focused design and multi-stage voltage multipliers. The current work’s rectifier demonstrates an optimization that contributes to the efficiency observed in the system’s performance.

The novelties in our design include the harmonic balance integrated with the rectenna design, facilitated by the incorporation of MA4E2054B1-1146T Schottky diodes. This innovation distinguishes our work from reference [36], which utilizes ambient Wi-Fi for power without a specific focus on harmonic integration. Reference [13] optimizes for RFID specific settings, whereas reference [37] emphasizes maximizing efficiency without noting specific diode implementation. Reference [9], although it presents a comprehensive system design, implemented only an HSMS286C diode into the rectifier design.

This benchmarking underscores the current study’s advancements in RF energy harvesting, marking a significant step forward in the field. The efficiency and output improvements, coupled with the novel integration of a harmonic balance technique, position our work as a state-of-the-art solution for RF energy-harvesting challenges, particularly for applications requiring high efficiency and compact design.

## 6. Conclusions

This paper presents the development of a rectenna prototype, employing RF energy harvesting for low-power RF-based applications. First, the rectifier comprises a seven-stage Cockroft–Walton voltage rectifier, which has been thoroughly investigated, simulated, fabricated, and tested in this study. The accumulation of parasitic capacitance from passive components and dielectric laminate constrains the cascading of stages in the rectifier, prompting the exploration of surface-mount and lumped-element devices for pre- and post-rectification. These components suppress higher harmonics and unwanted frequencies, enhancing the DC voltage output.

The optimal multiplication stage of rectifiers is determined through harmonic balance simulation. However, the limitation of simulation in accounting for parasitic influences with varying dielectric thickness diminishes the accuracy of the results. Simulated outcomes reveal that the optimal design for low-power Wi-Fi signals involves a 1.57 mm RO5880 substrate, a seven-stage rectifier with bandpass and low-pass filters. The MA4E2054B1-1146T Schottky diode demonstrates notable improvement over the HSMS286C, resulting in a forward-power increase of 95.0% over 25.54%, leading to a higher harvested DC voltage output. A 1.5 V threshold is achieved under a 1 MΩ load in real measurement tests with an RF generator, provided that rectifier prototype 1 achieves it at a 2 dBm power input. In comparison, prototype 2 sustains 1.5 V at a lower power input of −3 dBm.

The proposed fractal antenna has undergone comprehensive study, and the results of S_11_ and VSWR indicate its commendable transmission performance within a bandwidth below −10 dB. In simulation and measurement, reflected power is recorded at 91.40% and 97.99%, respectively. Despite the inability of the antenna to efficiently harvest Wi-Fi energy at long distances, its capacity to convert RF signals into AC has been demonstrated when connected to the rectifier, manifesting through the illumination of a red LED, indicating successful DC voltage harvesting at a distance of 15 mm for prototype 2.

Two configurations of the rectenna system have been proposed. The second rectenna prototype achieves a 1.5 V threshold at a 2 cm distance from the source based on results obtained with a Wi-Fi router as the RF power source. Moreover, the rectenna consistently maintains an average of 0.38 V at a distance of 26 cm, showcasing its superior performance to the first rectenna prototype. This paper concludes by advocating the utilization of the rectenna prototype for ultra-low-power RF-based applications.

## Figures and Tables

**Table 1:** Critical parameters of Schottky diodes.

Parameter	Description	Units	HSMS 2820	HSMS 2850	HSMS 2860	HSMS 286C	MA4E2054B1 1146T
Cj	Junction Capacitance	pF	0.7	0.18	0.18	0.18	0.13
Rs	Series Resistance	Ω	6	2.5	5	6	11

**Table 2:** Performance comparison of studied rectifiers.

Ref.	Substrate	Dielectric Thickness (mm)	Center Freq. (GHz)	Pin (mW)	R load (kOhm)	Vout (V)
[13]	RO5880	1.57	2.48	0.13	1000	2
[14]	RO5880	0.787	2.630	1.0	7.88	2.15
[15]	FR4	0.787	0.925	1.0	5.00	1.54
[16]	FR4	1.50	2.45	316	0.33	6.47

**Table 3:** Simulated DC voltage output with 0 dBm RF input power at 2.4 GHz.

Circuit Design	DC Voltage Output at 0 dBm (V)
1-stage rectifier circuit (HSMS286C)	2.2
1-stage rectifier circuit (MA4E2054B1-1146)	2.23
7-stage rectifier circuit	5.52
7-stage rectifier with LPF	5.98
8-stage rectifier circuit	9.32
8-stage rectifier circuit with LPF	9.48
Improved 7-stage rectifier circuit	7.27
Improved 7-stage rectifier circuit with LPF	7.802
Improved 7-stage rectifier with LPF and BPF	7.913
Improved 7-stage rectifier with LPF and BPF (MA4E2054B1-1146)	10.36
Improved 7-stage rectifier with LPF and BPF (FR4 and MA4E2054B1-1146T)	6.40

**Table 4:** Dimension of the fractal antenna.

L	W	L _M-K_	W _M-K_	L_f_	W_f_
40	25	23	0.66	16	1.8

**Table 5:** VSWR performance of rectifier prototypes.

	Rectifier Prototype 1	Rectifier Prototype 2
VSWR	13.59	1.57
Reflected Power (%)	74.46	4.92
Forward Power (%)	25.54	95.08

**Table 6:** Fractal antenna performances.

	Simulation	Fractal Antenna Prototype
VSWR	1.83	1.33
Reflected Power (%)	8.60	2.01
Forward Power (%)	91.40	97.99

**Table 7:** Measurement result of the signal antenna strength.

Antenna Distance (cm)	Fractal Antenna (dBm)
0	−4.06
5	−24.28
10	−21.38
15	−21.05
20	−17.56
25	−20.99
30	−21.56

**Table 8:** Rectifier performances with controlled RF power input.

	Prototype 1			Prototype 2		
Power Input (dBm)	Free Load (V)	1 M Ω Load (V)	Red LED Load (V)	Free Load (V)	1 M Ω Load (V)	Red LED Load (V)
21	23.61	23.19	1.96	33.91	32.66	1.71
20	22.39	21.81	1.94	32.75	31.53	1.70
19	20.76	20.04	1.92	31.86	30.87	1.69
18	18.15	17.51	1.90	31.16	30.01	1.69
17	16.24	15.71	1.89	30.29	28.45	1.68
16	14.48	14.02	1.87	28.38	26.04	1.67
15	13.10	12.71	1.85	26.37	23.94	1.66
14	11.70	11.31	1.83	24.51	22.09	1.65
13	9.95	9.53	1.81	23.16	19.88	1.64
12	8.68	8.23	1.80	19.14	17.12	1.63
11	7.63	7.15	1.78	16.22	14.45	1.62
10	6.70	6.21	1.76	13.71	12.16	1.61
9	5.75	5.24	1.74	11.27	9.95	1.59
8	5.29	4.79	1.73	10.18	8.96	1.58
7	4.33	3.824	1.70	7.93	6.98	1.55
6	3.79	3.264	1.67	6.77	5.87	1.52
5	3.25	2.723	1.63	5.68	4.82	1.48
4	2.76	2.233	1.58	4.74	3.95	1.43
3	2.35	1.846	1.52	4.01	3.29	1.37
2	2.01	1.515	1.47	3.44	2.75	1.31
1	1.84	1.345	1.43	3.13	2.45	1.28
0	1.54	1.065	1.37	2.63	2.01	1.20
−1	1.31	0.848	1.28	2.23	1.64	1.07
−2	1.11	0.670	1.13	1.90	1.33	0.90
−3	0.89	0.482	0.92	1.55	1.02	0.73
−4	0.75	0.376	0.76	1.30	0.80	0.59
−5	0.62	0.285	0.64	1.11	0.64	0.50
−6	0.53	0.216	0.54	0.93	0.50	0.43
−7	0.43	0.155	0.44	0.78	0.39	0.39
−8	0.40	0.114	0.38	0.66	0.29	0.40
−9	0.38	0.087	0.38	0.57	0.22	0.38
−10	0.37	0.066	0.38	0.39	0.17	0.39
−11	0.39	0.066	0.38	0.39	0.17	0.39
−12	0.38	0.066	0.38	0.38	0.17	0.38
−13	0.38	0.066	0.38	0.38	0.17	0.38
−14	0.38	0.066	0.38	0.38	0.17	0.38
−15	0.38	0.066	0.38	0.38	0.17	0.38
−16	0.38	0.066	0.38	0.38	0.17	0.38
−17	0.38	0.066	0.38	0.38	0.17	0.38
−18	0.38	0.066	0.38	0.37	0.17	0.38
−19	0.38	0.066	0.38	0.37	0.17	0.38
−20	0.38	0.066	0.38	0.37	0.17	0.38

**Table 9:** Distance required for LED to light up for rectenna prototype 1 and 2.

Rectenna Combination	Antenna Distance for Red LED to Light Up (mm)
Prototype 1	11
Prototype 2	15

**Table 10:** DC output (V) measurement of the rectenna system with Wi-Fi.

Distance between Antenna (cm)	Fractal Antenna with Prototype 1	Fractal Antenna with Prototype 2
0	5.710	18.12
1	1.731	7.79
2	0.672	3.312
3	0.390	1.184
4	0.356	0.410
5	0.276	0.393
6	0.225	0.374
7	0.187	0.369
8	0.176	0.364
9	0.158	0.348
10	0.151	0.323
11	0.130	0.381
12	0.080	0.418
13	0.070	0.396
14	0.054	0.389
15	0.052	0.388
16	0.050	0.369
17	0.040	0.357
18	0.032	0.370
19	0.021	0.375
20	0.020	0.365
21	0.025	0.234
22	0.040	0.188
23	0.060	0.144
24	0.060	0.180
25	0.035	0.390
26	0.021	0.395
27	0.016	0.325
28	0.018	0.193
29	0.026	0.115
30	0.030	0.054

**Table 11:** Ambient voltage reading in the rectenna system.

Rectenna Combination	DC Voltage Output (mV)
Prototype 1	2.9
Prototype 2	3.5

**Table 12:** Benchmarking of the current work with previous works.

Aspect	Current Work	Reference [36]	Reference [13]	Reference [37]	Reference [9]
Ultimate aim	Evaluating the integration of fractal antenna and RF energy harvester through simulation and experiment	Investigating the rectenna architecture for RFID through qualitative assesment	Examining the rectenna architecture with different multiplier topologies	Evaluating different antenna structures between monopole and fractal antenna	Evaluating different rectifier design for RF power energy harvester.
Measured output	3.53 V at −10 dBm input, scalable efficiency improvements	2 V with 2.4 GHz at −9 dBm input signal	2 V with 2.4 GHz at −4 dBm input signal.	Varied outputs based on design with maximum of 0.097 V	2 V output voltage at −9 dBm
Rectifier configuration	7-stage rectifier with 1.57 mm RO5880 substrate and MA4E2054B1-1146T Schottky diode compared with HSMS286C diode	7-stage rectifier with 1.57 mm RO5880 substrate	7-stage rectifier with 1.57 mm RO5880 substrate	7-stage rectifier with 1.57 mm RO5880 substrate	7-stage rectifier with 1.57 mm RO5880 substrate with HSMS286C diode
Novelties	Integration of harmonic balance and rectenna design with the introduction of MA4E2054B1-1146T Schottky diode	Utilization of ambient Wi-Fi for power	Optimization for RFID specific settings	Focused on maximizing efficiency	Comprehensive system design with different substrate

## Data Availability

Data are contained within the article.
